# Supplementary material for: High-Level Production of scFv-Fc Antibody Using an Artificial Promoter System with Transcriptional Positive Feedback Loop of Transactivator in CHO Cells
Source: Cells. 2023 Nov 16;12(22):2638. doi: 10.3390/cells12222638 (PMC10670205; doi:10.3390/cells12222638)
Supplement: Supplementary file 1 [file cells-12-02638-s001.zip › cells-2694565-supplementary.pdf]

Supplementary Materials

High-Level Production of scFv-Fc Antibody Using an Artificial Promoter System with Transcriptional Positive Feedback Loop of Transactivator in CHO Cells

Binbin Ying, Yoshinori Kawabe, Feiyang Zheng, Yuki Amamoto and Masamichi Kamihira*

Department of Chemical Engineering, Faculty of Engineering, Kyushu University, 744 Motooka, Nishi-ku Fukuoka 819-0395, Japan; ying.binbin.538@s.kyushu-u.ac.jp (B.Y.); kawabe@chem-eng.kyushu-u.ac.jp (Y.K.); zfylyhzw@gmail.com (F.Y.); amamoto.yuki.518@m.kyushu-u.ac.jp (Y.A.)

* Correspondence: kamihira@chem-eng.kyushu-u.ac.jp; Tel.: +81 92 802 2743

Supplementary Figure Legends

Figure S1. Flow cytometry analysis of GFP expression in CHO/aTF_GFP cells. (a) During cultivation at normal temperature (37°C) (turned "on" state). (b) After 8 days of Dox treatment. (c) After transient transfection of pCMV/aTF plasmid into Dox-treated cells.

Figure S2. Average values of scFv-Fc, glucose, and lactate in the culture supernatant obtained from semi-continuous culture of CHO/aTF_scFv-Fc2 cells. (a) scFv-Fc concentration. (b) scFv-Fc-specific production rate. (c) Glucose concentration. (d) Lactate concentration.

Figure S3. Scatter plots from DNA microarray analysis of CHO/aTF_scFv-Fc2 cells in semi-continuous culture. (a) Global gene expression signal values between each seeding cell density condition. (b) Signal values for genes that were upregulated with Z-scores of 5 or higher during low-temperature culture under all seeding cell density conditions.

Figure S4. Genes obtained from characteristic functional clustering analysis in DNA microarray analysis of CHO/aTF_scFv-Fc2 cells in semi-continuous culture. List of upregulated genes with the most significant differences in "Biological process" and "Cellular component."

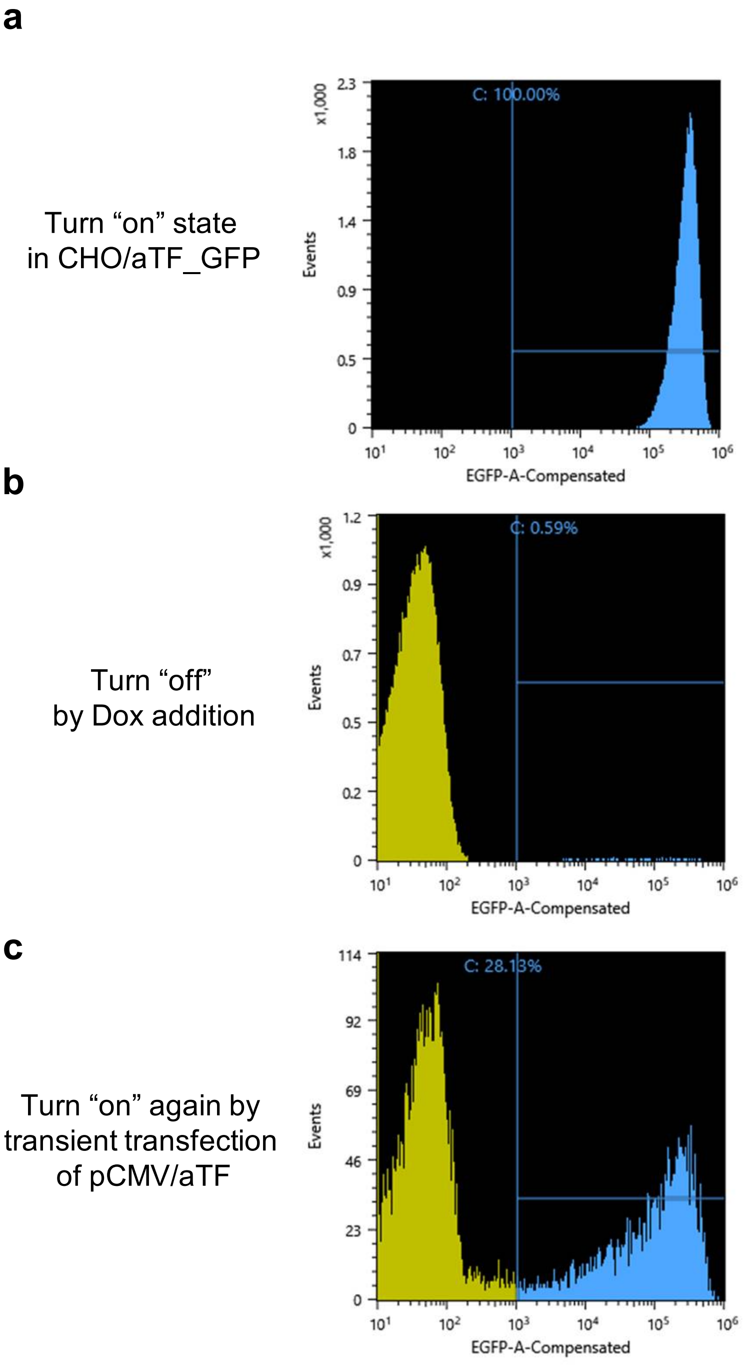

Figure S1. Flow cytometry analysis of GFP expression in CHO/aTF_GFP cells. (a) During cultivation at normal temperature (37°C) (turned “on” state). (b) After 8 days of Dox treatment. (c) After transient transfection of pCMV/aTF plasmid into Dox-treated cells.

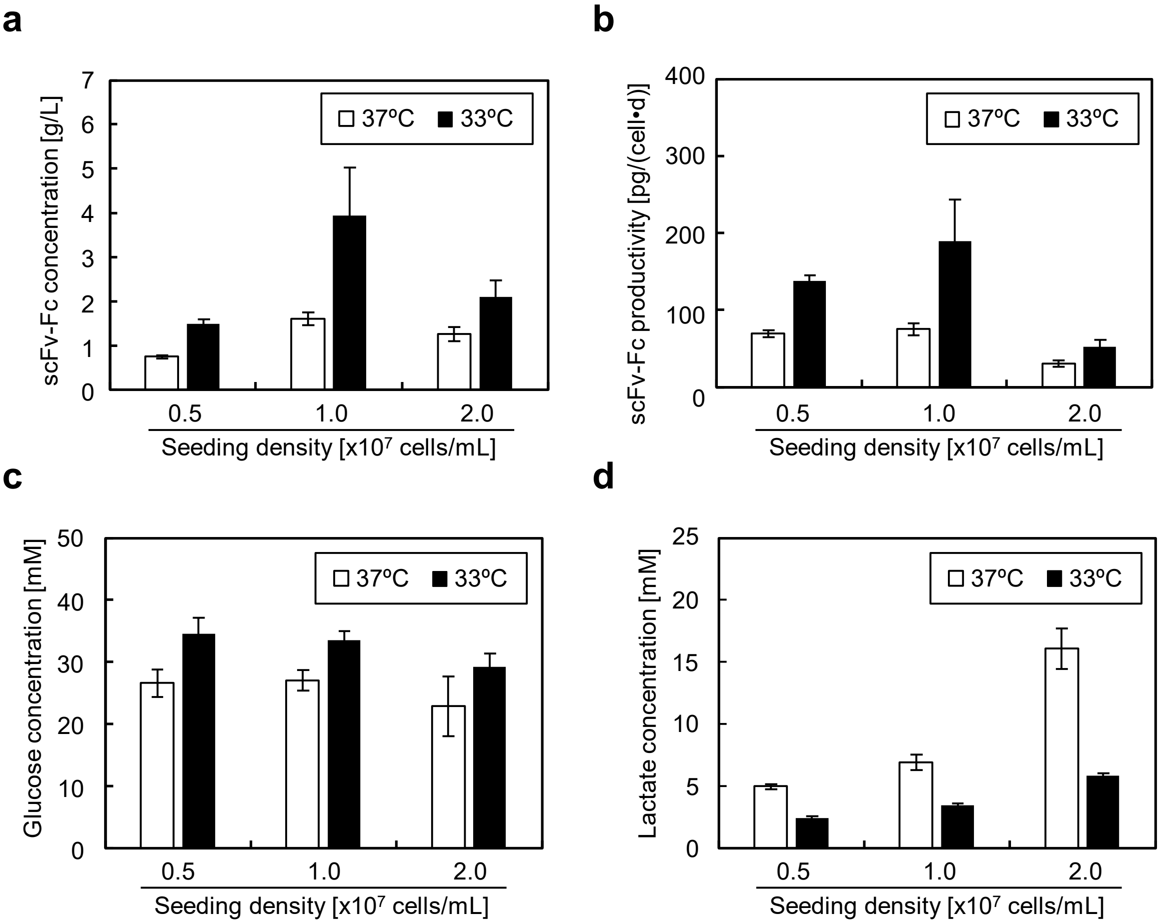

Figure S2. Average values of scFv-Fc, glucose, and lactate in the culture supernatant obtained from semi-continuous culture of CHO/aTF_scFv-Fc2 cells. (a) scFv-Fc concentration. (b) scFv-Fc-specific production rate. (c) Glucose concentration. (d) Lactate concentration.

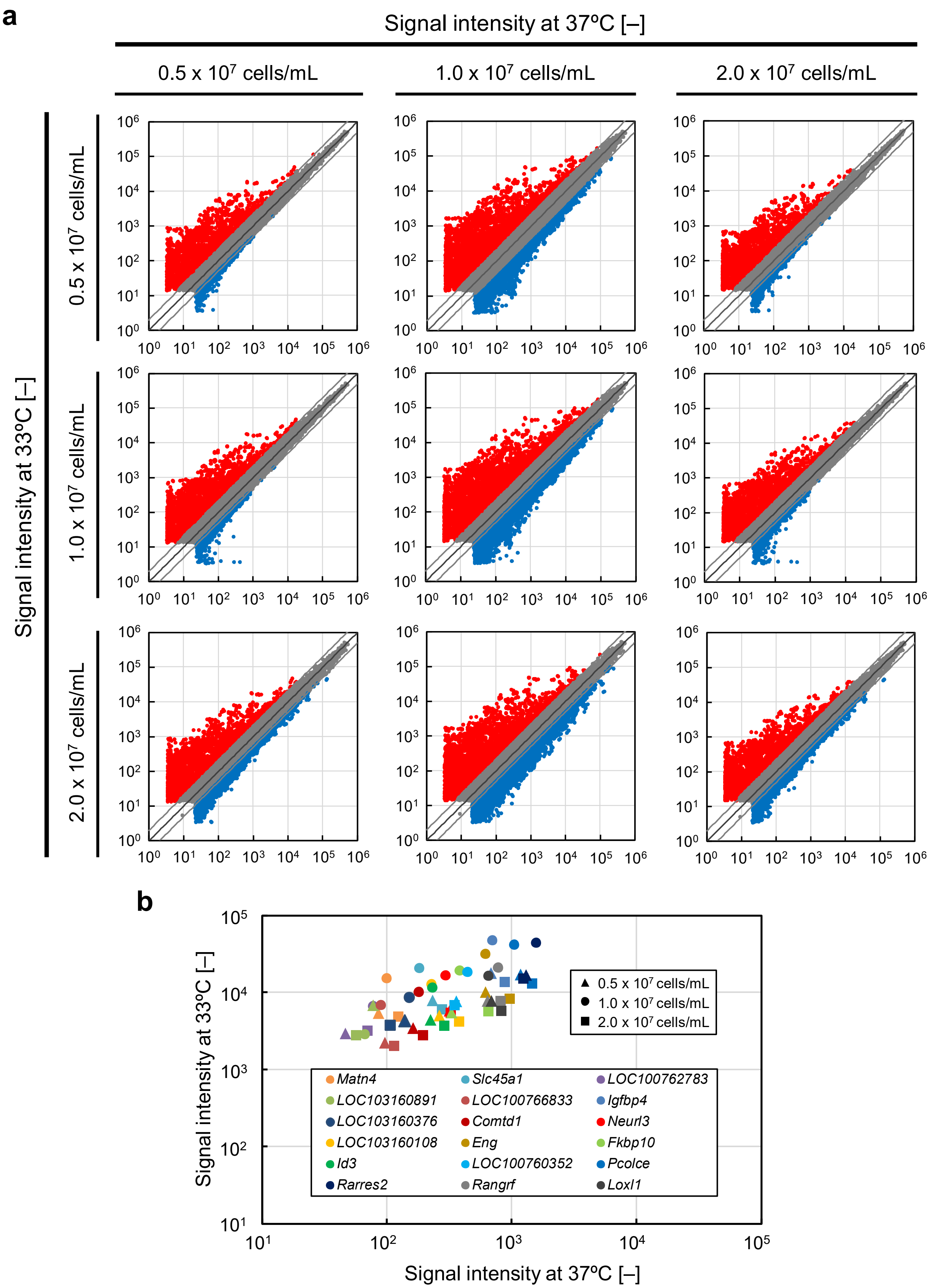

Figure S3. Scatter plots from DNA microarray analysis of CHO/aTF_scFv-Fc2 cells in semi-continuous culture. (a) Global gene expression signal values between each seeding cell density condition. (b) Signal values for genes that were upregulated with Z-scores of 5 or higher during low-temperature culture under all seeding cell density conditions.

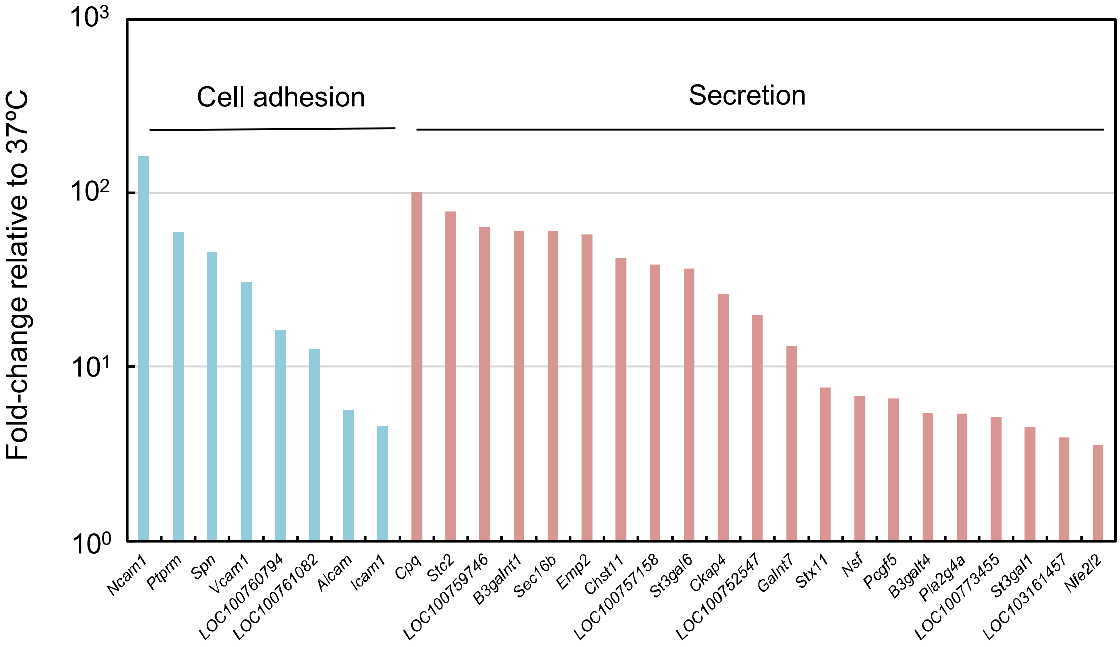

Figure S4. Genes obtained from characteristic functional clustering analysis in DNA microarray analysis of CHO/aTF_scFv-Fc2 cells in semi-continuous culture. List of upregulated genes with the most significant differences in "Biological process" and "Cellular component."

Figure S4

Table S1 Primer and probe lists for evaluation of transgene copy number by quantitative PCR

Target	Oligo type#	Sequence (5'→3')
**GFP**	F	CGAGGACAGCGTGATCTTC
	R	CCACGGTGGCGTTGCT
	P	CCGACAAGATCATCC
**scFv-Fc$**	F	AGTGCTGCACCAGGATTGG
	R	CTTGTTGGACACCTTGCACTTG
	P	TGAACGGCAAAGAGT
**scFv-Fc**	F	TGGAGTGGGAGAGCAATGG
	R	CGGGAGGCGTGGTCTTG
	P	AGCCGGAGAACAACT

#F, Forword primer; R, Reverse primer; P, Taqman probe. The probes were labeled with FAM dye 5' end and minor groove binder as a quencher at 3' end.
$scFv-Fc gene in donor vector plasmid using Cre-RMCE was codon-optimized based on human codon usage.

Table S2. Up-regulated genes in low temperature culture at 33ºC

Gene symbol	Gene description	Fold-change (*Z*-score)		
		0.5 x 10^7 cells/mL	1.0 x 10^7 cells/mL	2.0 x 10^7 cells/mL
Matn4	matrilin 4	61.78 (9.73)	154.23 (6.45)	39.16 (7.64)
Slc45a1	solute carrier family 45, member 1	33.46 (8.29)	114.80 (8.47)	21.65 (6.41)
LOC100762783	synaptonemal complex protein 3-like	45.42 (9.01)	86.76 (5.71)	61.83 (5.59)
LOC103160891	synaptonemal complex protein 3-like	43.64 (5.22)	85.26 (5.69)	48.57 (5.27)
LOC100766833	cytochrome b-245 light chain	22.81 (7.38)	77.54 (5.57)	17.41 (5.95)
Igfbp4	insulin-like growth factor binding protein 4	25.50 (12.92)	68.03 (7.54)	15.45 (7.39)
LOC103160376	late cornified envelope protein 1C-like	31.22 (8.12)	57.14 (5.18)	35.44 (7.43)
Comtd1	catechol-*O*-methyltransferase domain containing 1	20.78 (7.16)	57.04 (5.17)	14.29 (5.54)
Neurl3	neuralized E3 ubiquitin protein ligase 3	17.89 (6.81)	56.84 (7.22)	16.67 (5.86)
LOC103160108	multivesicular body subunit 12B	18.72 (6.92)	56.54 (5.16)	11.04 (5.01)
Eng	endoglin	16.20 (11.11)	51.49 (7.04)	8.64 (5.82)
Fkbp10	FK506 binding protein 10, 65 kDa	16.29 (6.59)	51.14 (7.03)	8.76 (5.86)
Id3	inhibitor of DNA binding 3, dominant negative helix-loop-helix protein	19.20 (6.98)	49.98 (5.01)	12.61 (5.28)
LOC100760352	serpin B6	20.79 (7.17)	42.12 (6.69)	19.25 (6.16)
Pcolce	procollagen C-endopeptidase enhancer	14.19 (10.58)	39.88 (6.59)	8.88 (5.90)
Rarres2	retinoic acid receptor responder (tazarotene induced) 2	12.83 (10.18)	28.49 (7.18)	12.11 (6.74)
Rangrf	RAN guanine nucleotide release factor	11.99 (9.91)	27.22 (5.91)	9.44 (6.06)
Loxl1	lysyl oxidase-like 1	11.14 (9.62)	25.43 (5.79)	6.95 (5.24)
